# Letter to the Editor

**DOI:** 10.1120/jacmp.v3i3.2569

**Published:** 2002-06-01

**Authors:** Paul Keall, Jeffrey Siebers

**Affiliations:** ^1^ Department of Radiation Oncology Virginia Commonwealth University Box 980058 Richmond VA 23298

## Abstract

PACS number(s): 87.90.+y, 87.52.–g

Dear Editor,

We read with interest the recent article by Wang *et al*. “Dosimetric advantage of using 6 MV over 15 MV photons in conformal therapy of lung cancer: Monte Carlo studies in patient geometries,” [J. Appl. Clin. Med. Phys. 3 (1), 51–59 (2002)]. In the abstract, the authors state that “In treatment planning of tumors that abut lung tissue, lower energy (6 MV) photon beams should be preferred over higher energies (15–18 MV) because of the significant loss of lateral dose equilibrium for high‐energy beams in the low‐density medium.” We would like to discuss this statement. In order to justify such a strong statement, one would expect (i) an accurate dose calculation algorithm to have been used for the treatment planning and plan evaluation, and (ii) a statistically significant number of cases to have been studied.

(i) In their paper, Wang *et al*. perform the treatment planning using a pencil beam (PB) method and evaluate their plans using a Monte Carlo (MC) method. Because PB agrees more closely with MC at low energies, the plan evaluation using MC at high energies has poorer target coverage. If MC had been used for the treatment planning, the poor target coverage would have been observed, and then improved by setting field apertures that account for the increased electron range. Obviously, larger apertures mean that more of the lung is in the field, however, this is the necessary depth dose versus lateral spread trade‐off as the beam energy changes. Planning and evaluating the plans with the same (accurate) method at least ensures a fair comparison.

(ii) The conclusions of Wang *et al*. were based on the treatment plans of two patients. With this sample size, it is feasible to hypothesize that lower energies are better than higher energies and perhaps state that a larger study would be useful. However, it is improper to conclude that lower energies are superior without proper statistical analysis. Drawing a general conclusion based on only the analysis of two patients is speculative (though over‐generalizing is something many of us are guilty of).

Our experience using IMRT to compare low‐ and high‐energy beams for lung cancer radiotherapy conflicts with the statement above. In our work, lung IMRT plans were calculated at both 6 and 18 MV using an algorithm (superposition) that accounts for the increased electron range in low‐density media. The IMRT objective function score was better for the 18 MV plan than the 6 MV plan. The 18 MV plan was used for treatment.

In summary, what Wang *et al*. have shown in their paper are discrepancies between treatment plans calculated by an MC method and those calculated by a PB algorithm. These discrepancies are larger for higher energy beams. Perhaps the statement above could be changed to: In treatment planning of tumors that abut lung tissue, in the absence of a dose calculation algorithm that fully accounts for the lateral electron range in low‐density media, lower energy (6 MV) photon beams should be preferred over higher energies (15–18 MV) to ensure target coverage.

